# Real-World Evidence Supporting Tandem Control-IQ Hybrid Closed-Loop Success in the Medicare and Medicaid Type 1 and Type 2 Diabetes Populations

**DOI:** 10.1089/dia.2022.0206

**Published:** 2022-10-31

**Authors:** Gregory P. Forlenza, Anders L. Carlson, Rodolfo J. Galindo, Davida F. Kruger, Carol J. Levy, Janet B. McGill, Guillermo Umpierrez, Grazia Aleppo

**Affiliations:** ^1^ Barbara Davis Center, Division of Pediatric Endocrinology, Department of Pediatrics, University of Colorado Denver, Denver, Colorado, USA.; ^2^ International Diabetes Center, HealthPartners Institute, Minneapolis, Minnesota, USA.; ^3^ Division of Endocrinology, Metabolism, and Lipids, Emory University School of Medicine, Atlanta, Georgia, USA.; ^4^ Division of Endocrinology, Diabetes, Bone and Mineral, Henry Ford Health System, Detroit, Michigan, USA.; ^5^ Division of Endocrinology, Diabetes, and Metabolism, Icahn School of Medicine at Mount Sinai, New York City, New York, USA.; ^6^ Division of Endocrinology, Metabolism and Lipid Research, Washington University in St. Louis, School of Medicine, St. Louis, Missouri, USA.; ^7^ Division of Endocrinology, Metabolism Emory University School of Medicine, Atlanta, Georgia, USA.; ^8^ Division of Endocrinology, Metabolism and Molecular Medicine, Feinberg School of Medicine, Northwestern University, Chicago, Illinois, USA.

**Keywords:** Type 1 diabetes, Type 2 diabetes, Hybrid closed loop, Special populations, Disparities

## Abstract

**Background:**

The Tandem Control-IQ (CIQ) system has demonstrated significant glycemic improvements in large randomized controlled and real-world trials. Use of this system is lower in people with type 1 diabetes (T1D) government-sponsored insurance and those with type 2 diabetes (T2D). This analysis aimed to evaluate the performance of CIQ in these groups.

**Methods and Materials:**

A retrospective analysis of CIQ users was performed. Users age ≥6 years with a t:slim X2 Pump and >30 days of continuous glucose monitoring (CGM) data pre-CIQ and >30 days post-CIQ technology initiation were included.

**Results:**

A total of 4243 Medicare and 1332 Medicaid CIQ users were analyzed among whom 5075 had T1D and 500 had T2D. After starting CIQ, the Medicare beneficiaries group saw significant improvement in time in target range 70–180 mg/dL (TIR; 64% vs. 74%; *P* < 0.0001), glucose management index (GMI; 7.3% vs. 7.0%; *P* < 0.0001), and the percentage of users meeting American Diabetes Association (ADA) CGM Glucometrics Guidelines (12.8% vs. 26.3%; *P* < 0.0001). The Medicaid group also saw significant improvement in TIR (46% vs. 60%; *P* < 0.0001), GMI (7.9% vs. 7.5%; *P* < 0.0001), and percentage meeting ADA guidelines (5.7% vs. 13.4%; *P* < 0.0001). Patients with T2D and either insurance saw significant glycemic improvements.

**Conclusions:**

The CIQ system was effective in the Medicare and Medicaid groups in improving glycemic control. The T2D subgroup also demonstrated improved glycemic control with CIQ use. Glucometrics achieved in this analysis are comparable with those seen in previous randomized controlled clinical trials with the CIQ system.

## Introduction

Hybrid closed-loop (HCL) systems combining an insulin pump, continuous glucose monitor (CGM), and automated control algorithm have demonstrated significant improvements in glycemic control in both randomized controlled and real-world observational trials.^1–5^ The Tandem t:slim X2 Control-IQ (CIQ) system (Tandem Diabetes, San Diego, CA) combines the t:slim X2 insulin pump, the Dexcom G6 CGM (Dexcom, San Diego, CA), and the CIQ HCL control algorithm to reduce hypo- and hyperglycemia and maximize CGM time in target range (TIR; 70–180 mg/dL).^6,7^ A National Institutes of Health (NIH)-sponsored randomized controlled trial of CIQ demonstrated significant improvement of +11% TIR compared to a sensor augmented pump (SAP) control group over 6 months among adults and adolescents with type 1 diabetes (T1D).^1^

Similar TIR improvement of +11% was demonstrated among children 6–13 years of age in a similar NIH-sponsored randomized controlled trial.^2^ Real-world analysis of 9451 CIQ users demonstrated attainment of 74%–75% TIR over 12-months of CIQ.^3^ Subanalysis of this cohort focused on those with the highest baseline glucose values demonstrated a +27% improvement in TIR among those with baseline glucose management indicator (GMI) ≥9% and a +28% improvement in TIR among those with baseline GMI ≥10%.^8^

Despite these noted benefits of HCL technology, there have been many barriers to technology adoption, particularly among people with insulin-requiring type 2 diabetes (T2D), Medicare beneficiaries and those covered by Medicaid insurance.^9–11^ Lower socioeconomic status, presence of government-sponsored insurance, and minority racial-ethnic status have been associated with lower rates of technology use (both insulin pump and CGM) and higher hemoglobin A1c (HbA1c) values in numerous studies.^9,10,12–15^ Evidence of the benefits of insulin pump therapy in T2D has been growing, with consistent improvements in HbA1c, reduction of hypoglycemia as well as total daily dose of insulin, both short and long term.^16–23^ In the Medicare and Medicaid beneficiaries in particular, coverage criteria determined by the Centers for Medicare and Medicaid Services currently limit access to insulin pumps, which contribute to these disparities.^24,25^

Furthermore, within Medicare beneficiaries, significant race-ethnicity-associated differences exist, and greater gaps in diabetes technology adoption have been noted.^11^ In this real-world retrospective analysis, we aimed to assess glycemic control outcomes with CIQ use among Medicare and Medicaid-beneficiaries with any type of diabetes and those with T2D with either type of insurance.

## Methods and Materials

### Study design/data sources

We performed a retrospective analysis of users of CIQ technology in the United States who had descriptive data available in Tandem's Customer Relations Management database and who had uploaded their glycemic data—either through Tandem's t:connect uploader or through the mobile app—to Tandem's t:connect web application from January 11, 2020 to January 11, 2022. The data extracted for analysis were deidentified. Participants consented to the use of their data for research purposes as part of their onboarding to Tandem when initiating their t:connect account. Glycemic outcomes were calculated for all participants who had at least 30 days of CGM data with ≥75% CGM availability before and after CIQ initiation.

The outcomes were calculated by participant and were limited to data collected while the participants were using Software versions 6 (t:slim X2 with Basal-IQ) and Software version 7 (t:slim X2 with CIQ). CGM values outside the valid ranges of 40–400 mg/dL were filtered out, with glucose value <40 and >400 mg/dL being saturated at 40 and 400 mg/dL, respectively. No CGM interpolation was performed. Glycemic outcomes are reported regardless of closed-loop status. No Institutional Review Board approval was sought for this retrospective analysis.

### Study population/participants

Data from users who were aged 6 years and older, had at least 12 consecutive months of data available on CIQ, and had at least 30 days of ≥75% CGM data availability before and after CIQ initiation were included in the analysis. Any CIQ users with data uploaded in t:connect that did not meet these criteria were excluded.

### Outcome metrics

Our primary outcome of interest was difference in TIR after starting CIQ use for at least 30 days after CIQ initiation, compared to baseline. We also analyzed change in GMI, time below range (TBR) 54–<70 mg/dL, TBR <54 mg/dL, time above range (TAR) >180 mg/dL, and TAR >250 mg/dL.

### Definitions

CGM metrics are calculated and presented as recommended by the international consensus on time in range and using the American Diabetes Association (ADA) targets for TIR metrics.^26^ Percent time in closed-loop automation was calculated as the percentage of the total basal rates delivered by the pump, in 5-min increments, which were decided by the CIQ algorithm.

Metrics for analysis were defined as follows:

- Time in range (TIR): Median of the % time spent in the clinically defined range of 70–180 mg/dL.
- TBR: Median of the % time spent in the clinically defined range of <70 mg/dL.
- Time in automation: Median percent of the total basal rates delivered by the pump, in 5-min increments, which were decided by the CIQ algorithm.
- GMI^27^: GMI = 3.31 + 0.02392 × [mean glucose in mg/dL]. The average glucose is calculated over the entire time a customer used a Tandem pump in accordance with the guidelines above.
- User Meeting ADA guidelines for CGM glucometrics^28^: GMI Below 7% with a Time in Range of >70% and either TBR of <4% for those younger than the age of 65 years or a TBR of <1% for those of age 65 years and older.

#### Exposure variables

Descriptive data were organized according to diabetes type, age, gender, time since diabetes diagnosis, GMI, previous Tandem pump software versions, and time in CIQ technology automation.

#### Definitions

- Prior HbA1c (Reported HbA1c): The last recorded HbA1c reported by a customer as part of the sales process. Values were included if they were taken within 6 months of initial t:connect data.
- Diabetes type: Self-reported clinical diabetes type 1 or type 2.
- Age: Age of customer at the report run-time.
- Sex: Self-reported sex
- Payer: The last payer the customer used for a Tandem pump or supply purchase available in Tandem's Customer Relations Management database.

### Statistical analysis

Outcomes were analyzed using Wilcoxon signed rank test and are reported as median (quartiles). Differences in meeting ADA guidelines for CGM glucometrics^28^ were analyzed using a test of proportions. Significance of differences in Time in Range and GMI were tested using Wilcoxon tests. Differences in meeting ADA guidelines were analyzed using a test of proportions.

## Results

### Study population

There were 5575 CIQ users with sufficient pre- and post-CIQ data for analysis (Table 1). The Medicare beneficiaries group consisted of 4243 CIQ users, with a mean ± standard deviation (SD) age of 67.4 ± 10.9 years, 48.3% male, of which 89% had T1D, 11% had T2D, and median (interquartile range [IQR]) baseline GMI was 7.3% (6.9%–7.7%). The Medicaid group consisted of 1332 CIQ users with a mean ± SD age of 22.3 ± 14.2 years, 44.1% male, of which 98% had T1D, 2% had T2D, and median (IQR) baseline GMI was 7.9% (7.4%–8.6%). The combined T2D subgroup with Medicare and/or Medicaid insurance consisted of 500 CIQ users with a mean ± SD age of 69.2 ± 10.5 years, 55.2% male, and with a median (IQR) baseline GMI of 7.3% (6.9%–7.7%).

**Table 1.** Cohort Baseline Characteristics

	All	Insurance cohorts		Type 2 diabetes (Medicare + Medicaid)
		Medicare	Medicaid	
*n*	5575	4243	1332	500
Mean age (years)	56.7 ± 22.5	67.4 ± 10.9	22.3 ± 14.2	69.2 ± 10.5
Male (%)	47.3	48.3	44.1	55.2
Diabetes type (%)				
Type 1	91	89	98	0
Type 2	9	11	2	100
GMI (%)	7.4 (7.0–7.9)	7.3 (6.9–7.7)	7.9 (7.4–8.6)	7.3 (6.9–7.7)
Mean SG (mg/dL)	171.0	166.8	191.9	166.8
TIR 70–180 mg/dL (%)	60	64	46	64
TBR 54–69 mg/dL (%)	0.75	0.74	0.74	0.26
TBR <54 mg/dL (%)	0.12	0.13	0.15	0.04
TAR 181–250 mg/dL (%)	28	26	27	27
TAR >250 mg/dL (%)	9.9	8	21	7

GMI, glucose management indicator; SG, sensor glucose; TAR, time above range; TBR, time below range; TIR, time in target range.

The cohort with Medicaid insurance was then subdivided by age group using standard age divisions: 6–13, 14–18, 19–64, and 65+ years. The 6–13 years age group (*n* = 394) was 10.4 ± 2.2 years of age and 49.5% male. The 14–18 years age group (*n* = 367) was 15.8 ± 1.4 years of age and 50.1% male. The 19–64 years age group (*n* = 554) was 33.7 ± 12.4 years of age and 35.6% male. The 65+ group (*n* = 17) was 69.0 ± 4.4 years of age and 64.7% male.

### Glycemic control in the Medicare cohort

**FIG. 1.**
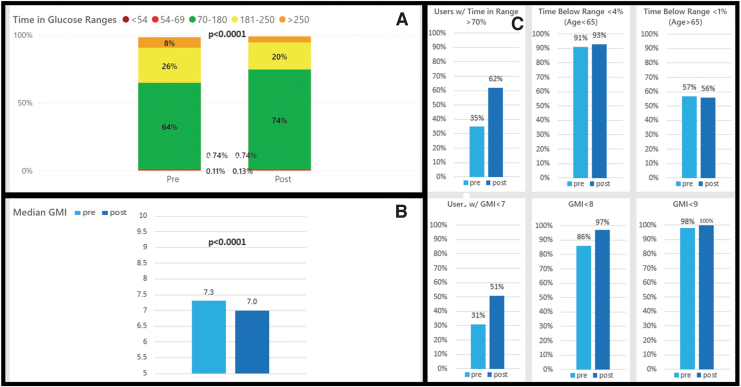
Medicare CIQ users pre- and post-CIQ glycemic metrics. **(A)** Change in time in ranges pre- and post-CIQ, *P*-value shown for pre-/postchange in TIR 70–180 mg/dL. **(B)** Change in GMI pre- and post-CIQ. **(C)** Percent of people meeting ADA TIR target, level 2 hypoglycemia target, level 1 hypoglycemia target, GMI <7%, 8%, and 9% targets. ADA, American Diabetes Association; CIQ, Control-IQ; GMI, glucose management indicator; TIR, time in target range.

Sufficient data were available for 4243 CIQ users who listed Medicare as their insurance. This cohort was 67.4 ± 10.9 years of age. After starting CIQ, the Medicare group saw a significant decrease in mean sensor glucose (SG) from 166.8 to 154.3 mg/dL (*P* < 0.0001), a significant decrease in GMI from 7.3% to 7.0% (*P* < 0.0001), and a significant increase in TIR from 64% to 74% (*P* < 0.0001; Table 2). This was seen with no change in level 1 hypoglycemia (0.74% vs. 0.74%; *P* = 0.327) and with a statistically significant, although overall minimal change in level 2 hypoglycemia (0.11% vs. 0.13%; *P* < 0.0001; Fig. 1).

**Table 2.** Glucose Management Indicator and Time in Range Using Tandem Control-IQ by Cohorts

	Medicare			Medicaid			Type 2 diabetes		
	Pre-CIQ	Post-CIQ	*P*	Pre-CIQ	Post-CIQ	*P*	Pre-CIQ	Post-CIQ	*P*
GMI (%)	7.3	7.0	<0.0001	7.9	7.5	<0.0001	7.3	7.1	<0.0001
Mean SG (mg/dL)	166.8	154.3	<0.0001	191.9	175.2	<0.0001	166.8	158.4	<0.0001
TIR 70–180 mg/dL (%)	64	74	<0.0001	46	60	<0.0001	64	72	<0.0001
TBR 54–69 mg/dL (%)	0.74	0.74	0.327	0.74	0.75	0.518	0.26	0.28	0.719
TRR <54 mg/dL (%)	0.11	0.13	<0.0001	0.15	0.18	<0.0001	0.04	0.06	<0.0001
TAR 181–250 mg/dL (%)	26	20	<0.0001	27	24	<0.0001	27	22	<0.0001
TAR >250 mg/dL (%)	8	5	<0.0001	21	13	<0.0001	7	5	<0.0001
Users meeting ADA goals (%)									
All guidelines	12.8	26.3	<0.0001	5.7	13.4	<0.0001	19.4	30.2	<0.0001
GMI <7%	30.6	50.9	<0.0001	10.3	18.1	<0.0001	28.8	40.8	<0.0001
TIR >70%	35.1	62.1	<0.0001	11.0	22.3	<0.0001	37.4	54.8	<0.0001
TBR <4%^*^	91	93	0.551	92	93	0.306	98	97	0.936
TBR <1%^**^	57	56	0.694	53	41	0.616	81	81	0.999

ADA, American Diabetes Association; CIQ, Control-IQ.

^*^ TBR of < 4% for those younger than the age of 65 years.

^**^ TBR of <1% for those of age 65 years and older.

At baseline, 12.8% of the Medicare cohort was meeting all ADA CGM guidelines for GMI, TIR, and TBR while after CIQ use, this percentage significantly increased to 26.3% (*P* < 0.0001). The percentage of users meeting the individual guideline for GMI <7% significantly increased from 30.6% to 50.9% (*P* < 0.0001). The percentage meeting the TIR goal of >70% also significantly increased from 35.1% at baseline to 62.1% with CIQ use (*P* < 0.0001). The percentage meeting TBR goals of <4% for age <65 years and <1% for age ≥65 years remained unchanged (Table 2).

Within the Medicare cohort, there were 806 users who transitioned from multiple daily injection (MDI) therapy to CIQ therapy. This subgroup had a higher baseline GMI at 7.9% and saw a significant decline in GMI to 7.1% (difference of −0.8%; *P* < 0.0001).

### Glycemic control in the Medicaid cohort

**FIG. 2.**
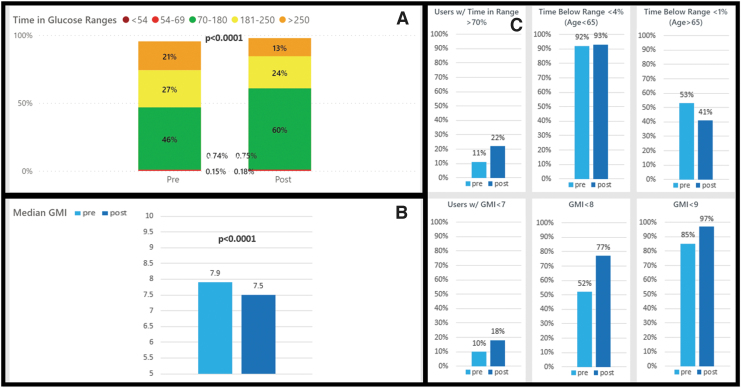
Medicaid CIQ users pre- and post-CIQ glycemic metrics. **(A)** Change in time in ranges pre- and post-CIQ, *P*-value shown for pre-/postchange in TIR 70–180 mg/dL. **(B)** Change in GMI pre- and post-CIQ. **(C)** Percent of people meeting ADA TIR target, level 2 hypoglycemia target, level 1 hypoglycemia target, GMI <7%, 8%, and 9% targets.

Sufficient data were available for 1332 users who listed Medicaid as their insurance. After starting CIQ, the Medicaid population saw a significant decrease in mean SG from 191.9 to 175.2 mg/dL (*P* < 0.0001), a significant decrease in GMI from 7.9% to 7.5% (*P* < 0.0001), and a significant increase in TIR from 46% to 60% (*P* < 0.0001) (Table 2). This was seen with no change in level 1 hypoglycemia (0.76% vs. 0.72%; *P* = 0.518) and with a statistically significant, although overall minimal change in level 2 hypoglycemia (0.15% vs. 0.18%; *P* < 0.0001; Fig. 2).

At baseline, only 5.7% of the Medicaid cohort was meeting all the ADA CGM guidelines for GMI, TIR, and TBR, while after CIQ use this number more than doubled to 13.4% (*P* < 0.0001). The percentage of users meeting the individual guideline for GMI <7% significantly increased from 10.3% to 18.1% (*P* < 0.0001) while the percentage meeting the TIR goal of >70% more than doubled from 11.0% to 22.3% (*P* < 0.0001). The percentage meeting TBR goals (levels 1 and 2) remained unchanged.

**FIG. 3.**
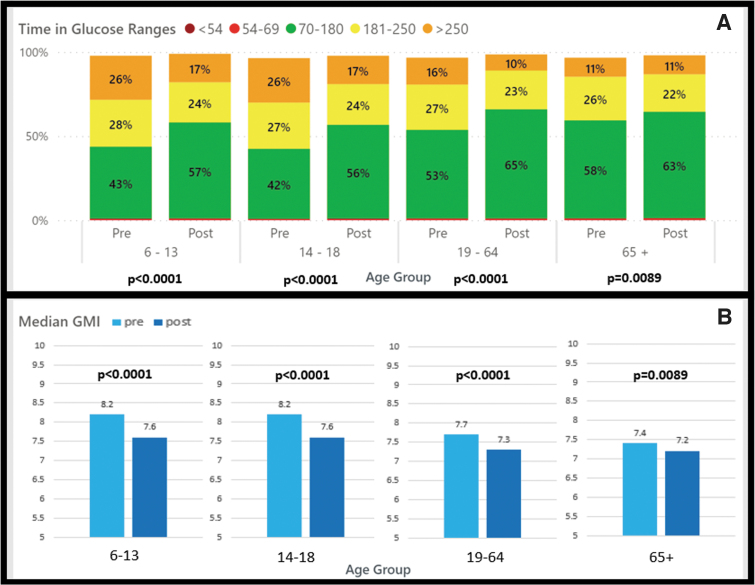
Medicaid CIQ users pre- and post-CIQ analysis by age group. **(A)** Change in time in ranges pre- and post-CIQ by age group, *P*-values shown for pre-/postchange in TIR 70–180 mg/dL by age group. **(B)** Change in GMI pre- and post-CIQ.

The Medicaid cohort was further subanalyzed by age group (Table 3). The age-group based analysis demonstrated that significant improvement for GMI was seen for children 6–13 years of age (8.2% vs. 7.6%; *P* < 0.0001), adolescents 14–18 years of age (8.2% vs. 7.6%; *P* < 0.0001), adults 19–64 years of age (7.7% vs. 7.3%; *P* < 0.0001), and seniors 65+ years of age (7.4% vs. 7.2%; *P* = 0.0089). Across all age groups TIR was also significantly increased without significant change in level 1 or level 2 hypoglycemia (Fig. 3).

**Table 3.** Glucose Management Indicator and Time in Range Using Tandem Control-IQ among Medicaid Patients by Age Group

	6–13 years			14–18 years			19–64 years			65+ years		
	Pre-CIQ	Post-CIQ	*P*	Pre-CIQ	Post-CIQ	*P*	Pre-CIQ	Post-CIQ	*P*	Pre-CIQ	Post-CIQ	*P*
GMI (%)	8.2	7.6	<0.0001	8.2	7.6	<0.0001	7.7	7.3	<0.0001	7.4	7.2	0.0089
Mean SG (mg/dL)	204.4	179.4	<0.0001	204.4	179.4	<0.0001	183.5	166.8	<0.0001	171.0	162.6	0.0089
TIR 70–180 mg/dL (%)	43	57	<0.0001	42	56	<0.0001	53	65	<0.0001	58	63	0.0011
TBR 54–69 mg/dL (%)	0.72	0.81	0.0281	0.65	0.69	0.4642	0.84	0.80	0.1655	0.83	1.06	0.7119
TRR <54 mg/dL (%)	0.14	0.19	0.0008	0.13	0.18	<0.0001	0.16	0.18	0.0174	0.21	0.17	0.3627
TAR 181–250 mg/dL (%)	28	24	<0.0001	27	24	<0.0001	27	23	<0.0001	26	22	0.0008
TAR >250 mg/dL (%)	26	17	<0.0001	26	17	<0.0001	16	10	<0.0001	11	11	0.0056
Users meeting ADA goals (%)												
All guidelines	2.8	7.6	0.0023	3.5	9.3	0.0015	9.2	20.6	<0.0001	5.9	5.9	0.9999
GMI <7%	5.9	10.4	0.0189	6	10.9	0.0169	15.9	27.8	<0.0001	23.5	35.3	0.4516
TIR >70%	5.6	11.4	0.0033	6.3	15	<0.0001	15.9	27.8	<0.0001	23.5	35.3	0.4516
TBR <4%^*^	92	93	0.5792	94	96	0.1600	90	90	0.7640	n/a	n/a	n/a
TBR <1%^**^	n/a	n/a	n/a	n/a	n/a	n/a	n/a	n/a	n/a	53	41	0.4920

^*^ TBR of < 4% for those younger than the age of 65 years.

^**^ TBR of <1% for those of age 65 years and older.

The percentage of users meeting all ADA guidelines for CGM glucometrics was significantly improved in the 6–13-, 14–18-, and 19–64-year age groups and unchanged in the 65+-year age group. The percentage meeting the individual guideline for GMI <7% and TIR >70% was significantly improved in the 6–13-, 14–18-, and 19–64-year age groups, and unchanged in the 65+-year age group (Table 3).

Within the Medicaid group, there were 659 users who transitioned from MDI therapy to CIQ therapy. This group had a baseline GMI of 8.6% and saw a significant decline in GMI to 7.4% (difference of −1.2%; *P* < 0.0001).

### Glycemic control among Medicaid and Medicare CIQ users with T2D

**FIG. 4.**
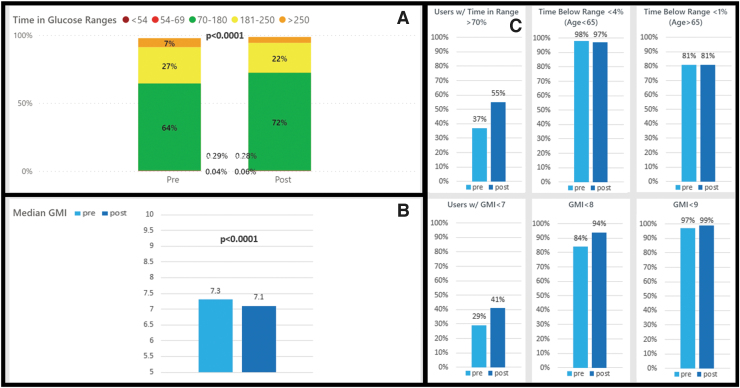
Medicare + Medicaid CIQ users with type 2 diabetes pre- and post-CIQ glycemic metrics. **(A)** Change in time in ranges pre- and post-CIQ, *P*-value shown for pre-/postchange in TIR 70–180 mg/dL. **(B)** Change in GMI pre- and post-CIQ. **(C)** Percent of people meeting ADA TIR target, level 2 hypoglycemia target, level 1 hypoglycemia target, GMI <7%, 8%, and 9% targets.

There were 500 individuals with Medicare or Medicaid insurance who listed their diabetes category as T2D. The population with T2D saw a significant decline in mean SG from 166.8 to 158.4 mg/dL (*P* < 0.0001), a significant decrease in GMI from 7.3% to 7.1% (*P* < 0.0001), and a significant increase in TIR from 64% to 72% (*P* < 0.0001) (Table 2). This was seen without a change in level 1 hypoglycemia and with a small, but statistically significant change in level 2 hypoglycemia (0.04%–0.06%; *P* < 0.0001) (Figure 4).

The percentage of people with T2D meeting all ADA CGM guidelines for GMI, TIR, and TBR significantly increased with CIQ use from 19.4% to 30.2% (*P* < 0.0001). For the individual guidelines, there was significant improvement in the percentage meeting ADA CGM guidelines for GMI and TIR with CIQ use. The percentage meeting guidelines for TBR (levels 1 and 2) did not change among the T2D cohort.

Among the T2D subgroup, there were 238 users who transitioned from MDI therapy to CIQ therapy. They had a baseline GMI of 8.1% and saw a significant improvement in GMI to 7.2% (difference of −0.9%; *P* < 0.0001).

## Discussion

The CIQ system was effective in the Medicare and Medicaid beneficiaries' groups, including a subset of patients with T2D, at improving glycemic control as assessed by a variety of glycemic metrics and target goals. There was significant reduction in GMI in the Medicare group by 0.3%, in the Medicaid group by 0.4%, and among the T2D subset by 0.2%. There was also significant improvement in TIR in the Medicare group by 10%, in the Medicaid group by 14%, and in the T2D subset by 8%. These improvements were seen without significant change in level 1 hypoglycemia in any of the analyses, although with slight increases in level 2 hypoglycemic exposure. There were also significant improvements in the percentage of people meeting ADA CGM guidelines for all targets, GMI, and TIR for all the groups analyzed.

The CIQ system has previously been tested in a large NIH-sponsored series of randomized controlled trials.^1,2^ The data published by Brown et al., demonstrated that compared to SAP, CIQ use resulted in an 11% improvement in TIR, 0.33% reduction in HbA1c, 13 mg/dL reduction in average sensor glucose, and 0.88% reduction in level 1 hypoglycemia.^1^ In that study, CIQ users reached a TIR of 71%, HbA1c of 7.06%, and TBR % <70 mg/dL of 1.58%. In the present analysis, users with Medicare saw a similar decline in HbA1c reaching a similar value of about 7.0%.

They also saw a similar improvement in TIR reaching a slightly higher TIR of 74%. This was done while maintaining a TBR % <70 mg/dL of under 1%. There was a statistically significant, although clinically insignificant, increase in level 2 hypoglycemia from 0.11% to 0.13%. These findings demonstrate that the Medicare beneficiaries group is seeing similar benefits with CIQ use to those seen in the Tandem t:slim with CIQ pivotal study by Brown et al.^1^

The Medicaid cohort contained pediatric, adolescent, and adult participants. Among the 14–18-, 19–64-, and 65+-year old Medicaid CIQ users, GMI and TIR were significantly improved across all age groups. This was seen without a change in level 1 hypoglycemia. There was a statistically significant, although likely clinically insignificant, increase in level 2 hypoglycemia from 0.15% to 0.18%. The GMI reductions were −0.6%, −0.4%, and −0.2%, respectively. The TIR improvements were +14%, +13%, and +5%, respectively. The changes for the age cohorts of 14–18 and 19–64 year also compare favorably with the improvements seen in the study by Brown et al.^1^ The improvements seen in the 65+-year cohort were somewhat smaller, although still statistically significant within this analysis.

The data published by Breton et al., compared SAP use to CIQ use in an NIH-sponsored randomized controlled trial.^2^ In this pediatric study, CIQ use resulted in an 11% improvement in TIR, 0.4% reduction in HbA1c, 13 mg/dL reduction in average glucose, and no change in % <70 mg/dL. In that study, CIQ users reached a TIR of 67%, HbA1c of 7.0%, and TBR % <70 mg/dL of 1.6%. The 6–13-year-old CIQ Medicaid users saw a GMI improvement of 0.6% reaching a GMI of 7.6% with a TIR improvement of 14% reaching a TIR of 57%, and with a TBR % <70 mg/dL below 1%. These results demonstrate that the real-world pediatric Medicaid population is showing a similar or even greater benefit from CIQ use compared to that seen in the NIH-trial by Breton et al.^2^

The evidence on insulin pump therapy benefits in T2Ds and older adults with T2Ds, such Medicare age beneficiaries, continues to increase. A multicenter open-label, clinical trial with 56 T2D participants (mean age 57 ± 10 years) studied the effects of insulin pump initiation after discontinuation of all other oral medications except for metformin. After 16 weeks, the mean HbA1c decreased by 1.2 ± 1.2% (*P* < 0.001) with no episodes of severe hypoglycemia.^16^

The Opt2mise Glucose Control in Type 2 Diabetes Mellitus With Insulin Pump Therapy (OpT2mise) was the first large scale randomized trial that studied the effects of insulin pump therapy in 331 adults with T2D, including 69 subjects aged 65 years and older. In this trial, the decrease in HbA1c was greater in the insulin pump group compared to the MDI group with a between-group treatment difference of −0.7% (95% confidence interval −0.9 to −0.4; *P* < 0.0001) and a 21% reduction of mean total daily insulin dose in the pump treatment group (*P* < 0.0001).^17^

In addition, durable effects of insulin pump therapy in T2D have been reported as well. A cohort of 161 T2D (mean age at insulin pump start 58.3 ± 9.8 years) was followed for up to 9 years (mean follow-up 5.1 ± 3.2 years). After 1 year, the HbA1c had decreased by 1.3% from baseline (*P* < 0.001), and after 9 years of follow-up, the HbA1c decrease was maintained (*P* < 0.05).^29^

Very recently, a retrospective observational study of 3952 T2D participants using a tubeless insulin pump showed a reported reduction in mean HbA1c by −1.3 ± 1.7% (*P* < 0.0001) after 90 days of use. Within this cohort, 914 participants (25%) were aged 65 years or older, and they also experienced a decrease in HbA1c of −0.9% ± 1.3 (*P* < 0.0001).^23^

Finally, an open label, randomized, crossover pilot study compared glucose control under single hormone HCL versus MDI in adults with T2D older than the age of 55 (mean age 63.6 ± 6.7 years old). In this short study, TIR improved by 21.6% overnight and 7.3% over 24 h without increasing hypoglycemia risk when using HCL.^4^

Despite the growing evidence of clinical benefits of insulin pump therapy in T2D, the value of insulin pump therapy with HCL has been underestimated, potentially due to perceived overall less risk for hypoglycemia in this population if early in the disease and using newer classes of pharmacologic agents.^28^ However, older people with T2D such as those on Medicare, and with advanced disease requiring insulin, are a greater risk of hypoglycemia, not only due to insulin therapy but also due to the likely presence of diabetic complications such as renal disease.^30,31^ The analysis of a retrospective cohort study of 50,439 T2D subjects from 2006 to 2015 revealed that the incidence of severe hypoglycemia increased from 0.12% in 2006 to 0.31% in 2015 (*P* = 0.01) and the presence of severe hypoglycemia was higher in those with previous diagnosis of nonsevere hypoglycemia (9% vs. 2%, *P* < 0.001).^32^

Moreover, fear of hypoglycemia in the patient with T2D is a very real entity. A survey of 424 T2D participants, of which 53.3% on insulin, studied the hypoglycemic attitudes and behavioral scale. The insulin using group had longer duration of diabetes, higher HbA1c, history of severe hypoglycemia (all *P* < 0.001), and greater fear of hypoglycemia than the noninsulin using group (*P* < 0.001).^33^ Therefore, automated insulin delivery systems use in T2D present an excellent regimen modality to not only improve glucometrics, but also to reduce hypoglycemia regardless of the HbA1c levels.

As demonstrated by this real-world analysis of CIQ use, in the 500 individuals with Medicare or Medicaid who listed their diabetes category as T2D, the HbA1c significantly decreased from 7.3% to 7.1% (*P* < 0.001) with significant increase in TIR to 72% (*P* < 0.001), without changes in level 1 hypoglycemia and a small but statistically significant reduction of level 2 hypoglycemia (*P* < 0.001). In addition, in the 238 users who transitioned from MDI therapy to CIQ therapy, the GMI decreased from baseline by −0.9% (8.1%–7.2%, *P* < 0.001). CIQ users with T2D who met all the ADA CGM guidelines for GMI, TIR, and TBR increased to 30.2% (*P* < 0.001).

Strengths of the present study include the large sample sizes for all of the groups analyzed, which are significantly larger than those available for device prospective trials. The analysis also benefits from a real-world setting, which makes the data more generalizable to clinical practice. Weaknesses of the present study include the lack of follow-up biological HbA1c data and thus the reliance on GMI as a surrogate. The need to have uploaded device data may decrease the generalizability of results as those device users who did not upload their data would not be represented. The analyses were performed using a reporting dashboard of real-world data and are limited to predetermined analyses existing within the dashboard tools.

Other metrics of interest such as daytime versus nighttime analysis, mealtime analysis, analysis of coefficient of variation of sensor glucose, and analysis of newer metrics such as Glycemia Risk Index are not currently available within these tools.

In conclusion, the present analysis demonstrates that the use of CIQ HCL technology is highly successful at improving glycemic control in Medicare and Medicaid beneficiaries, including those with T2D when compared to their previous therapy. The glycemic targets achieved compares favorably in terms of GMI, mean SG, and TIR to the levels achieved in the HCL group within prior randomized controlled clinical trials of this system. These findings support our hypothesis that Medicare and Medicaid beneficiaries with both T1D and T2D benefit from the CIQ HCL system, and prescribers should utilize this technology to achieve glycemic benefits within these individuals.
